# Site-Specific Differences in T Cell Frequencies and Phenotypes in the Blood and Gut of HIV-Uninfected and ART-Treated HIV+ Adults

**DOI:** 10.1371/journal.pone.0121290

**Published:** 2015-03-26

**Authors:** Steven A. Yukl, Amandeep K. Shergill, Valerie Girling, Qingsheng Li, Maudi Killian, Lorrie Epling, Peilin Li, Philipp Kaiser, Ashley Haase, Diane V. Havlir, Kenneth McQuaid, Elizabeth Sinclair, Joseph K. Wong

**Affiliations:** 1 Department of Medicine, San Francisco VA Medical Center, San Francisco, California, United States of America; 2 Department of Medicine, University of California, San Francisco (UCSF), San Francisco, California, United States of America; 3 School of Biological Sciences, University of Nebraska-Lincoln, Lincoln, Nebraska, United States of America; 4 Department of Microbiology, University of Minnesota, Minneapolis, Minnesota, United States of America; Uniformed Services University, UNITED STATES

## Abstract

Gastrointestinal T lymphocytes are critical for mucosal immunity and HIV pathogenesis, yet little is known about normal T cell numbers and phenotypes in different regions of the gut, or the degree to which ART can restore levels to those of HIV-uninfected individuals. To investigate these questions, we measured T cell frequencies and markers of memory, activation, anergy, and homing in the blood, ileum, and rectum of HIV- and ART-suppressed HIV+ adults. In HIV- individuals, T cell frequencies and phenotypes differed significantly between sites. Compared to HIV- adults, HIV+ adults had lower absolute CD4+T cell counts in the ileal lamina propria and lower relative CD4+T cell counts in the blood and ileum. In the gut, HIV+ adults had a higher proportion of CD38+ CD4+T cells, a lower proportion of terminally-differentiated effector cells, and, in the rectum, a higher proportion of CTLA-4+ CD4+T cells. In HIV+ individuals, relative CD4+T cell numbers in the ileum correlated with the proportion of CTLA-4+ CD4+T cells, whereas in the rectum, they tended to correlate with the proportion of circulating CD4+T cells expressing α4β7 or CCR6. Mechanisms of T cell reconstitution may differ throughout the gut, with homing contributing more in the rectum while ileal reconstitution is associated with mucosal CD4+T cell anergy.

## Introduction

Gastrointestinal T lymphocytes are critical for mucosal immunity and play key roles in the pathogenesis of HIV as well as its ability to persist on antiretroviral therapy (ART). HIV infection causes massive depletion of CD4+T cells (>80%) in the gut [1,2,3,4,5,6], which occurs prior to [2,3] and exceeds [1,4,6] CD4+T cell depletion in the blood or lymphoid tissues. Though ART can raise peripheral CD4+T cell counts to the normal range, it is unclear whether ART can completely restore CD4+T cells in the gut [7]. While many studies have shown delayed[8,9] and incomplete restoration after ART [6,9,10,11,12,13,14], other studies have suggested that complete restoration could be achieved [9,15,16,17]. These studies differed in the timing of ART initiation, length of treatment, method of quantifying CD4+ cells (relative or absolute), and gut location sampled.

Little is known about the normal variation in T cell numbers and phenotypes throughout the GI tract [18]. Relatively few studies in treated HIV+ patients have examined more than one gut site [19,20,21,22,23,24,25], and few of these have included HIV- individuals[21,22,24]. In one study of ART-treated HIV+ patients, HIV levels and T cell frequencies varied significantly across the gut, with the ileum having the highest HIV transcriptional activity (RNA/DNA) and the rectum having the highest HIV DNA and CD4+T cell frequency[19]. The ileum may differ in other ways, as one study of ART intensification suggested that some patients on ART may have ongoing replication in the ileum but not other sites[20]. Unfortunately, relatively few studies have sampled the ileum, and only two included data on HIV- individuals[21,22].

Even less is known about CD4+T cell phenotypic variation throughout the gut, especially in the ileum and rectum. One area of uncertainty is the distribution of T cell maturation subsets throughout the gut. Central memory (CM) and transitional memory (TM) CD4+T cells are increasingly recognized as a major reservoir for HIV DNA in the blood[26], and effector memory (EM) cells may play a similar role in the gut[27]. Several studies have examined the distribution of these cells in the gut of HIV+ patients[11,14,24,27], but they disagreed as to whether most cells are CM[14] or EM[11,24,27]; only one presented data for HIV- subjects[14], and comparative data is lacking for the ileum and rectum.

Another area of uncertainty is the normal degree of T cell activation in the gut and the degree to which ART reverses HIV-associated changes. Although previous studies have measured the proportion of activated or cycling (Ki67+) T cells in the gut of ART-treated patients, relatively few have presented comparative data for HIV- individuals [10,11,17], they disagree as to whether ART restores normal numbers of HLA-DR+ T cells[11,17], and comparative data is unavailable for CD38 or for the ileum. Similarly, little is known about expression of the anergy/inhibitory receptor CTLA-4 in the gut of HIV+ or HIV- patients. In one study, the proportion of rectal CD4+T cells that expressed CTLA-4 or PD-1 was higher in untreated and treated HIV+ individuals compared to controls, and the mean fluorescence intensity of both markers correlated with plasma viral load[28]. No information is available for the ileum.

Likewise, little is known about the expression of homing receptors in the gut. The integrin α4β7 mediates homing of T cells to the gut, binds to the HIV envelope[29], triggers killing of uninfected CD4+T cells, and may mark cells that are preferentially infected with SIV[30,31]. Several studies have examined β7 expression in relation to gut immune reconstitution [10,21,32], and three have examined levels of β7 in the jejunum[9,10,32], but comparative data is lacking for other sites. CCR6 and CXCR3 also mediate homing of T cells to the gut and mark peripheral CD4+T cells that are preferentially infected with HIV[33], yet no data is available regarding the frequencies of cells expressing these markers in the gut of HIV-infected patients. Combinations of CXCR3, CCR4, and CCR6 can also be used to distinguish Th1, Th2, Th17, and Th1Th17 cells[34].

To address these gaps in knowledge, we measured T cell frequencies, maturation subsets, levels of T cell “activation” (CD38, HLA-DR), expression of CTLA-4, expression of homing receptors, and Th subsets in the blood, terminal ileum, and rectum of ART-suppressed HIV+ patients (n = 18) and HIV- comparators (n = 16). We hypothesized that uninfected persons would show differences in T cell frequencies and phenotypes in these three different sites, and that HIV-infected patients would show site-specific differences in the degree to which ART restores normal cell frequencies and phenotypes, with the least restoration in the ileum. As a first step to explore mechanisms of immune reconstitution, we also assessed for correlations between CD4+T cell frequencies and the proportion of CD4+T cells that express markers of activation, anergy, or homing to the gut.

## Materials and Methods

### Study Participants

HIV+ participants were recruited from San Francisco General Hospital (n = 4) and the San Francisco VA Medical Center (SFVAMC; n = 14). Inclusion criteria included: 1) age 18–65; 2) infection with HIV-1; 3) ART for ≥12 months prior to study entry; 4) no change in ART for ≥3 months prior to study entry; 5) CD4+ T cell count≥200 cells/μl; and 6) HIV RNA<50 copies/ml for ≥6 months prior to study entry. HIV- participants were recruited from the SFVAMC (n = 16). Inclusion criteria included: 1) age ≤65; 2) scheduled for screening colonoscopy; and 3) HIV- status as confirmed by review of the medical records and repeat HIV ELISA. Exclusion criteria included prior diagnosis or endoscopic evidence of inflammatory bowel disease, other active enterocolitis, or GI malignancy. The study was approved by the Committee on Human Research (CHR) of the University of California, San Francisco (UCSF) and the VA Clinical Research Workgroup of the SFVAMC. All participants provided written informed consent. Blood was obtained by venous phlebotomy immediately prior to colonoscopy.

### Colonoscopy and Biopsy

Colonoscopies were performed using an Olympus CF-H180 or PC-Q180AL colonoscope (Olympus America, San Jose, CA, USA). Biopsies were obtained from endoscopically normal appearing areas of the terminal ileum (when accessible) and rectum (4 biopsies/site in HIV- and 7–15 biopsies/site in HIV+ participants) using the Radial Jaw 3, 3.7mm maximum capacity forceps or the Radial Jaw 4, 2.8mm large capacity forceps (Boston Scientific, Marlborough, MA, USA). In a few individuals, the ileum could not be reached by colonoscopy (5/16 HIV-) or rectal biopsies were not obtained (3 other HIV-, 2/18 HIV+).

### Processing of Gut Biopsies

In a fraction of individuals (8 HIV+, 7 HIV-), one ileal biopsy was fixed in 4% paraformaldehyde for immunohistochemistry. The remaining biopsies from each site were combined in culture medium (RPMI with L-Glu, penicillin/streptomycin, and 15% fetal calf serum [R-15]), washed with R-15, and dissociated to total gut cells using 3 rounds of collagenase digestion, needle shearing, cell straining, and washing[19]. Cells from the three digestion rounds were combined, pelleted, resuspended in PBS+0.1% BSA+2mM EDTA (buffer A), and aliquoted for flow cytometry (0.5x10^6^ cells per panel).

### Processing of Blood

Blood was collected using 8.5ml BD Vacutainer ACD tubes with solution A (Becton, Dickinson, and Company, Franklin Lakes, NJ, USA), and peripheral blood mononuclear cells (PBMC) were isolated by centrifugation on Ficoll-Paque PLUS (G.E. Healthcare, Pittsburgh, PA, USA)[19], washed, resuspended in buffer A, counted, and aliquoted for flow cytometry (0.5x10^6^ cells per panel).

### Flow Cytometry

Cells from PBMC, ileum, and rectum were stained with Live/Dead Fixable Aqua Cell Stain (Life Technologies, Grand Island, NY, USA), blocked with human gamma globulin, stained with antibodies, and washed as described previously[27]. Panels for flow cytometry included the following monoclonal anti-human antibodies: Panel 1: CD45-APC (clone 2D1; 1:66.5), CD3-Pacific Blue (UCHT1; 2.5:66.5), CD38-PE (HB719; 0.4:66.5), and HLA-DR-FITC (L243; 5:66.5) (BD Biosciences, San Jose, CA, USA); CD4-ECD (SFCI12T4D11; 0.25:67; Beckman Coulter, Inc., Brea, CA, USA); CD8-QDOT605 (3B5; 0.05:67; Life Technologies); Panel 2: CD45RO-FITC (UCHL1; 2:67), CD3 Alexa Fluor700 (UCHT1; 2:67), CCR7 Alexa Fluor647 (150503; 2:67), CD152(CTLA-4)-PE (BNI3; 5:67) (BD Biosciences); CD4-ECD (SFCI12T4D11; 0.25:67; Beckman Coulter, Inc.); CD45-Pacific Blue (HI30; 2:67), CD8-QDOT605 (3B5; 0.05:67; Life Technologies); CD27 APC-eFluor780 (0323; 1:67; eBioscience, San Diego, CA, USA); Panel 3: CD45-FITC (2D1; 1:77.5), CD3 Alexa Fluor700 (UCHT1), integrin β7-PE-Cy5 (FIB504; 5:77.5), CCR4-V450 (1G1; 5:77.5), and CCR6-APC (11A9; 2.5:77.5) (BD Biosciences); CD4-ECD (SFCI12T4D11; 0.25:67; Beckman Coulter); CD8-QDOT605 (3B5; Life Technologies); CXCR3-PE (CXCR3–173; 4:77.5; BioLegend, San Diego, CA, USA). Panels 2 and 3 were developed over the course of the study, so data from these panels is available for a smaller subset of participants (Table 1).

**Table 1:** Clinical Characteristics.

Measure	Flow cytometry panel	HIV-	HIV+
Mean age (range) in years	1 (16 HIV-, 18 HIV+)	58.4 (50–65)	51.6 (33–64)
	2,3 (8 HIV-, 8HIV+)	61.9 (57–65)	54.6 (44–64)
Sex	1	All male	All male
	2,3	All male	All male
Race	1	White: 87.5%	White: 72.2%
		Black: 6.3%	Black: 16.7%
		Asian: 0%	Asian: 5.6%
		Hispanic: 6.3%	Hispanic: 5.6%
	2,3	White: 87.5%	White: 75%
		Black: 0%	Black: 25%
		Asian: 0%	Asian: 0%
		Hispanic: 12.5%	Hispanic: 0%
Time since HIV diagnosis (years)	1		17.8 (10–26)
	2,3		19.6 (12–26)
Nadir CD4 (cells/mm^3^)	1		210.7 (0–469)
	2,3		200.1 (0–430)
Total time on ART^1^ (years)	1		9.8 (1.3–16)
	2,3		10.7 (1.3–16)
Duration of viral suppression (yrs)	1		4.3 (1.1–12)
	2,3		3.5 (1.1–4.7)
CD4 count*(cells/mm^3^)	1		619.8 (289–1552)
	2,3		609.5 (467–1060)
CD4%*	1		31.7% (20–53%)
	2,3		31% (27–39%)
CD8 count*0020(cells/mm^3^)	1		835.1 (433–1422)
	2,3		844.3 (617–1422)
CD8%*	1		44.2% (25–61%)
	2,3		43.3% (35–57%)
ART Regimen^2^	1		FTC/TDF/EFV: 39%
			FTC/TDF/ATV/r: 22%
			ABC/3TC + PI(+/-r): 11%
			3 NRTI + PI/r: 17%
			2 NRTI + NNRTI +PI/r: 11%
	2,3		FTC/TDF/EFV: 50%
			FTC/TDF/ATV/r: 25%
			2–3 NRTI + LPV/r: 25%

^1^ART = Antiretroviral therapy.

^2^Abbreviations: FTC = emtricitabine; TDF = tenofovir; EFV = efavirenz; ATV/r = atazanavir/ritonavir ABC = abacavir; 3TC = lamivudine; PI = protease inhibitor (atazanavir, lopinavir, or fosamprenavir); r = ritonavir boosting; NRTI = nucleoside reverse transcriptase inhibitor; NNRTI = non-nucleoside reverse transcriptase inhibitor.

*CD4 and CD8 counts were measured by the clinical laboratory from samples collected immediately prior to endoscopy.

For panels 1 and 3, cells were fixed by resuspending in 200ul of 0.5% formaldehyde at 4C overnight and data was acquired the following day on a customized BD LSR II Flow cytometer. For panel 2, fresh cells were run the same day on a FACS Aria cytometer. Data was analyzed using Flowjo Software (Treestar, Inc., Ashland, OR, USA). Cells were sequentially gated by scatter (to identify single cells), CD45+ cells (leukocytes), and live CD3+ cells (T cells), then gated on CD4+ and CD8+ cells, then gated on other markers (S1 Fig., S2 Fig.). Gates were set using Fluorescent-Minus-One controls for each marker on a PBMC sample, and then applied to PBMC and gut samples from the same individual. Using panel 2, combinations of CD45RO, CCR7, and CD27 were used to categorize T cells into naïve, central memory, transitional memory, effector memory, “other memory,” or terminally-differentiated effector cells (Table 2, S1 Fig.).

**Table 2:** Markers used to Define CD4+T Cell Maturation Subsets.

	CD45 RO	CCR7	CD27
Naïve	−	+	+
Terminally differentiated	−	−	−
Central Memory	+	+	+
Effector Memory	+	−	−
Transitional Memory	+	−	+
“Other Memory”	+	+	−

### Immunohistochemistry (IHC)

Ileal biopsies from 8 HIV+ and 7 HIV- participants were fixed in 4% paraformaldehyde, though biopsies from 5 HIV- participants were lost in shipping. Immunohistochemistry for CD3 and/or CD4 detection was performed as described previously[35]. Primary anti-CD3 (SP7, 1:100, Lab Vision, Fremont, CA, USA) and anti-CD4 (IF6, 1:5 dilution, Novocastra Laboratories Ltd/Leica Biosystems, Buffalo Grove, IL, USA) antibodies and an EnVision System (DakoCytomation) with horseradish peroxidase as polymer conjugated enzyme and 3,39-diaminobenzidine as substrate were used on 6μm tissue sections. Quantification of absolute CD4+T cells/mm^3^ in the lamina propria and (where present) lymphoid aggregates was performed using a positive pixel count algorithm in Aperio’s Spectrum Plus analysis program (version 9.1; Aperio ePathology Solutions/Leica Biosystems) as described previously[36].

### Statistics

For each outcome measure, comparisons between anatomic sites in HIV- individuals were performed using the Wilcoxon signed rank test, while for a given site, results for HIV- and HIV+ individuals were compared using the Mann-Whitney test. Correlations were performed using the Spearman test. Statistics were calculated using GraphPad Prism 5.0.

## Results

### Clinical characteristics

Study participants were all males, with a mean age of 58.4 in the HIV- group and 51.6 in the HIV+ group (Table 1). The mean duration of HIV infection was 17.8 years.

### CD4 counts

**Fig 1:**
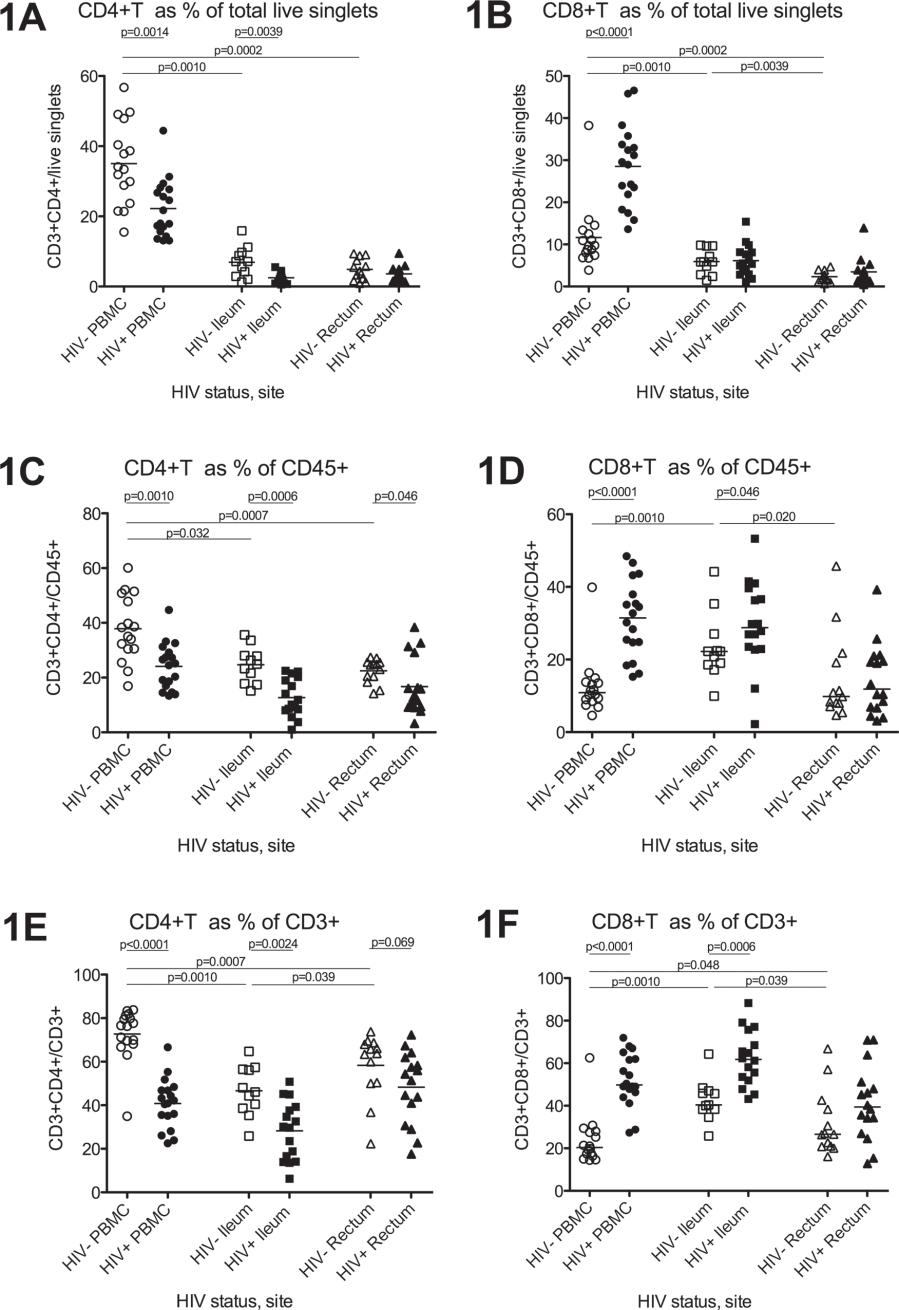
Relative CD4+T cell numbers in blood, ileum, and rectum. Flow cytometry was used to measure CD4+T cell (1A,C,E) and CD8+T cell (1B,D,F) frequencies as a percentage of all live singlet cells (1A-B), leukocytes (1C-D), and T cells (1E-F) in peripheral blood mononuclear cells (PBMC; circles), ileum (squares), and rectum (triangles) of HIV- (open shapes) and HIV+ (black shapes) participants. Bars indicate the mean.

Flow cytometry was used to measure the frequency of CD4+T cells (CD3+CD4+) as a percentage of: 1) total cells (live singlets); 2) leukocytes (CD45+); and 3) T cells (CD3+). Compared to HIV- participants, HIV+ participants had a lower ratio of CD4+T cells to total cells (CD4+T/total) in the PBMC and ileum but not rectum (Fig. 1A), and a lower ratio of CD4+T cells to leukocytes (CD4+T/CD45+) in all sites (1C).

In HIV- individuals, the ratio of CD4+T cells to all T cells (CD4%) differed significantly between blood, ileum, and rectum (1E), with the highest proportion in the blood and the next highest in the rectum. Compared to HIV- participants, HIV+ participants had a lower CD4% in the PBMC and ileum and a trend towards a lower CD4% in the rectum (1E). The ratios (HIV+/HIV-) of the mean CD4% were 0.56, 0.59, and 0.83 for blood, ileum, and rectum.

In the subset of individuals for whom fixed ileal biopsies were available, immunohistochemistry was used to measure the number of CD4+T cells per mm^3^ in lamina propria (effector site) and lymphoid aggregates (inductive site). Compared to HIV- participants, HIV+ participants tended to have fewer CD4+T cells in the lamina propria (S3 A,B Fig.). Since lymphoid aggregates were not observed in every individual, the sample size was insufficient to compare CD4+T cell counts in the lymphoid aggregates.

### CD8 counts

In HIV- individuals, the ratio of CD8+T cells to total cells (CD8+T/total) was greater in PBMC than ileum and greater in ileum compared to rectum (Fig. 1B), while CD8+T/CD45+ and CD8% were higher in ileum compared to either PBMC or rectum (1D,F). Compared to HIV- participants, HIV+ participants had: 1) higher CD8+T cell frequencies (as ratio of total cells, CD45+ cells, and T cells) in the PBMC (1B,D,F); and 2) higher CD8+T/CD45+ and CD8% but not CD8+T/total in the ileum (1D,F,B). No differences were detected in the rectum.

### T cell maturation subsets

**Fig 2:**
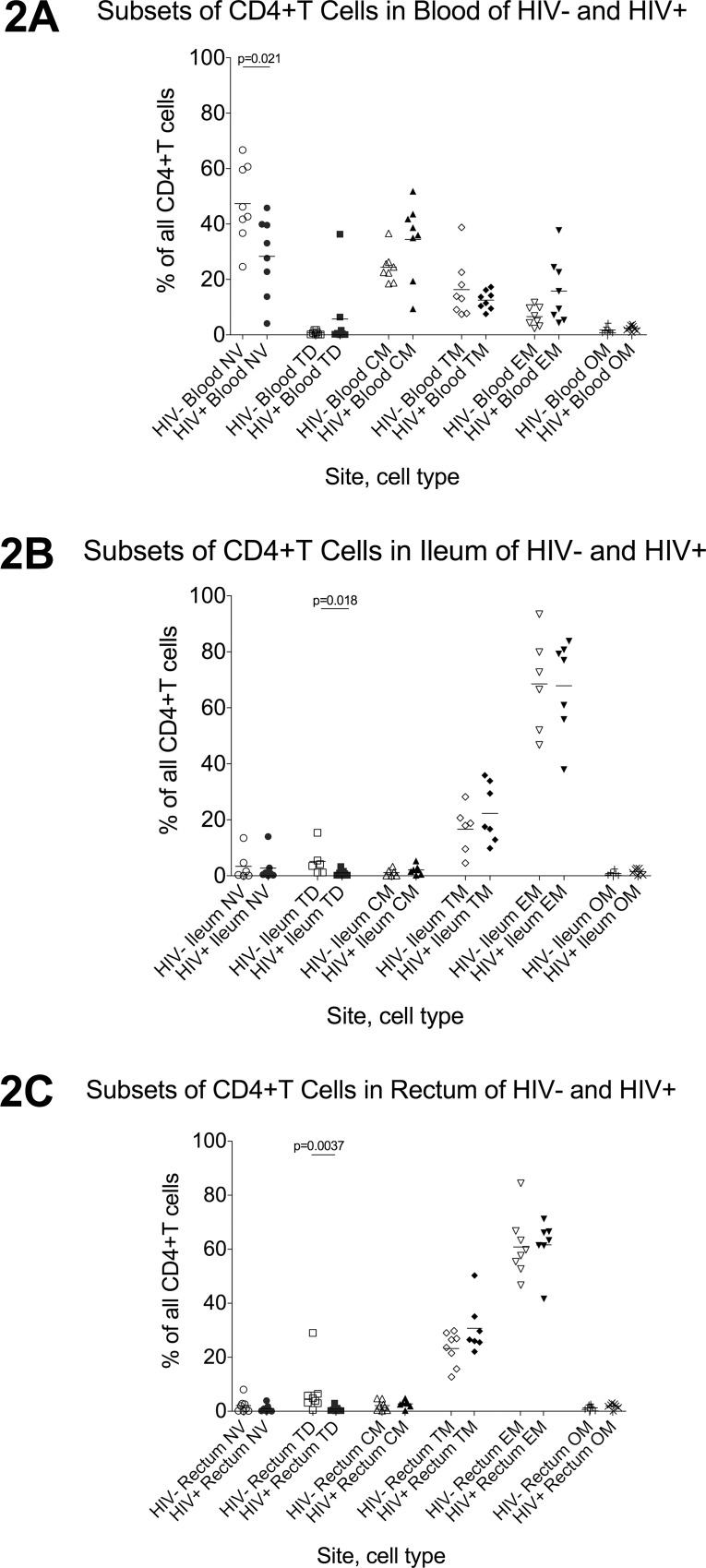
CD4+T cell maturation subsets in blood, ileum, and rectum. Using the combinatorial expression of CD45RO, CCR7, and CD27 (Table 2), flow cytometry was used to measure the proportion of all CD4+T cells in blood (2A), ileum (2B), and rectum (2C) that are naïve (NV; circles), terminally-differentiated (TD; squares), central memory (CM; triangles), transitional memory (TM; diamonds), effector memory (EM; inverted triangles), or “other memory” (OM; X’s) CD4+T cells in HIV- (open shapes) and HIV+ (black shapes) participants. Bars indicate the mean.

**Fig 3:**
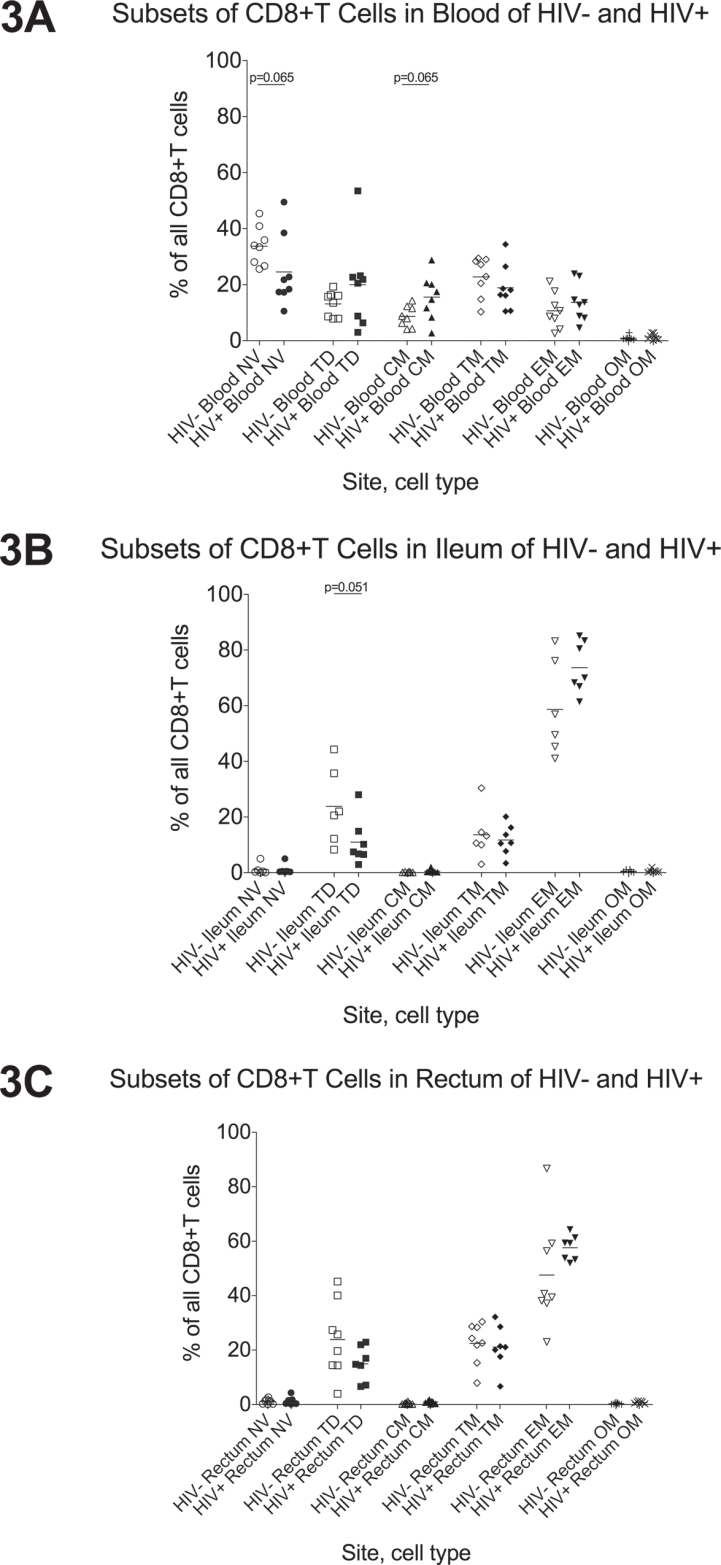
CD8+T cell maturation subsets in blood, ileum, and rectum. Flow cytometry was used to measure the proportion of all CD4+T cells in blood (3A), ileum (3B), and rectum (3C) that are naïve (NV; circles), terminally-differentiated (TD; squares), central memory (CM; triangles), transitional memory (TM; diamonds), effector memory (EM; inverted triangles), or “other memory” (OM; X’s) CD4+T cells in HIV- (open shapes) and HIV+ (black shapes) participants. Bars indicate the mean.

In HIV- individuals, naïve cells represented the largest proportion of CD4+ and CD8+T cells in the blood (Fig. 2A, Fig. 3A), while both gut sites had mostly effector and transitional memory T cells, with very low proportions of naïve, central memory, or terminally differentiated T cells (2B-C, 3B-C). Compared to HIV- participants, HIV+ participants had fewer naïve CD4+T cells (2A) and a trend towards fewer naïve CD8+T cells in the blood (3A). In the gut, HIV+ individuals had fewer terminally-differentiated CD4+T cells in both gut sites (2B-C) and a trend towards fewer terminally-differentiated CD8+T cells in the ileum (3B). No differences were observed in other subsets.

### T cell activation

**Fig 4:**
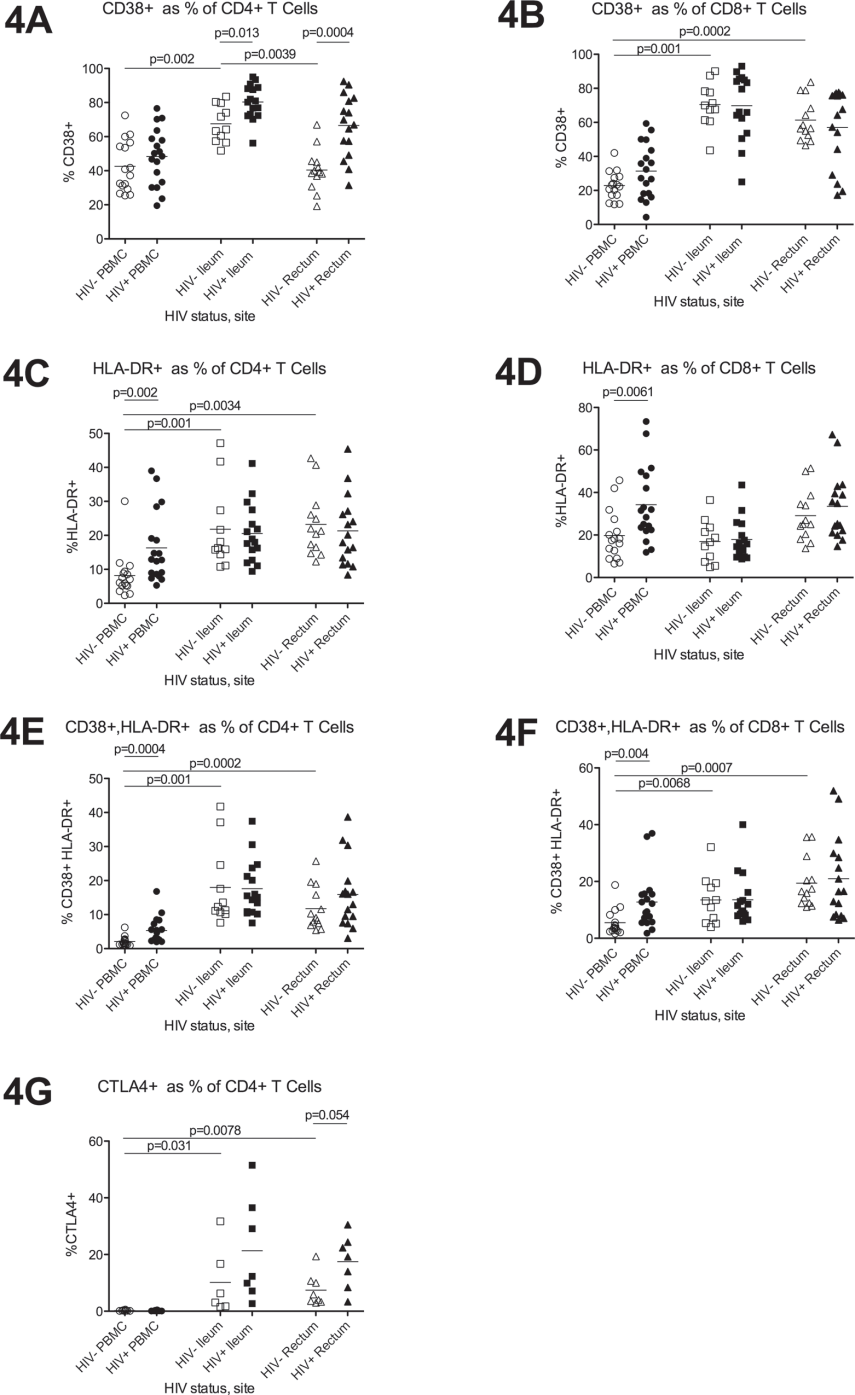
Expression of CD38, HLA-DR, and CTLA-4 in blood, ileum, and rectum. Flow cytometry was used to measure the proportion of all CD4+T cells (4A,C,E,G) and CD8+ T cells (4B,D,F) that express CD38 (4A-B), HLA-DR (4C-D), both CD38 and HLA-DR (4E-F), or CTLA-4 (4G) in the PBMC (circles), ileum (squares), and rectum (triangles) of HIV- (open shapes) and HIV+ (black shapes) participants. Bars indicate the mean.

In HIV- individuals, the proportion of all CD4+T cells that were CD38+ (Fig. 4A) was higher in ileum compared to either PBMC or rectum, while the proportions of HLA-DR+ (4C) and CD38+HLA-DR+ (4E) CD4+T cells were higher in both gut sites compared to PBMC. Compared to HIV- participants, HIV+ participants had a higher proportion of CD38+ CD4+T cells in both gut sites but not PBMC (4A), while the proportions of HLA-DR+ and CD38+HLA-DR+ CD4+T cells were higher in PBMC but not in either gut site (4C,4E).

In HIV- individuals, the proportions of CD8+T cells that were CD38+ (4B) and CD38+HLA-DR+ (4F) were higher in both gut sites compared to blood, while no differences between sites were observed in the proportions of HLA-DR+ CD8+T cells (4D). Compared to HIV- participants, HIV+ participants had a higher percentage of HLA-DR+ and CD38+HLA-DR+ CD8+T cells in the blood (4D,4F), while no differences were observed in CD38+ CD8+T cells in the blood, or in CD38+, HLA-DR+, or CD38+HLA-DR+ CD8+T cells in either gut site.

### CTLA-4

In HIV- individuals, the proportion of CD4+T cells that expressed CTLA-4 was considerably higher in both gut sites compared to the PBMC (Fig. 4G). Compared to HIV- participants, HIV+ participants tended to have a higher proportion of CTLA-4+ CD4+T cells in the rectum (p = 0.054), but no differences were observed in PBMC or ileum (4G).

### Homing markers

**Fig 5:**
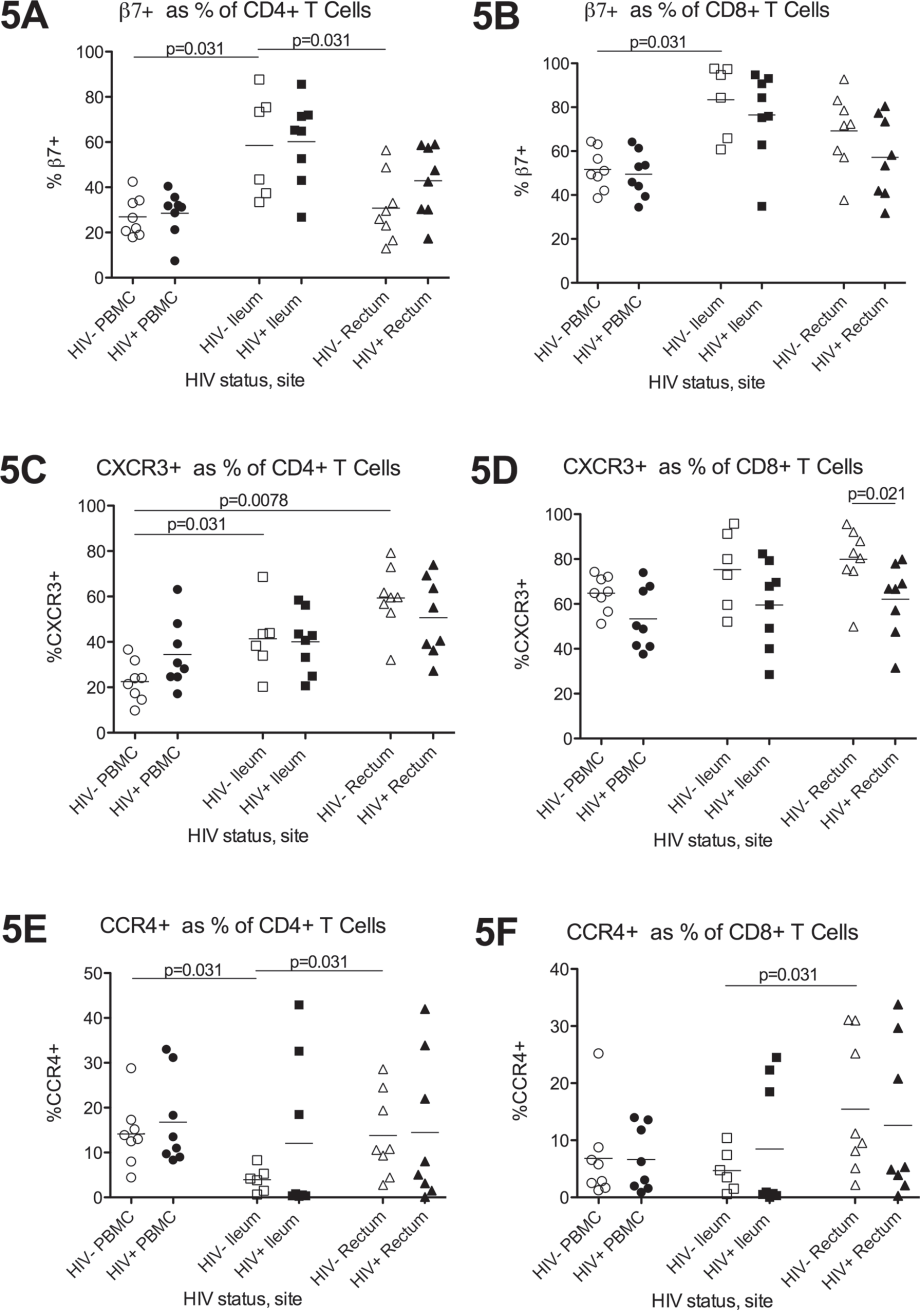
Expression of homing markers. Flow cytometry was used to measure the proportion of CD4+T cells (5A,C,E) and CD8+T cells (5B,D,F) that express β7 (5A-B), CXCR3 (5C-D), or CCR4 (5E-F) in PBMC (circles), ileum (squares), and rectum (triangles) of HIV- (open shapes) and HIV+ (black shapes) participants. Bars indicate the mean.

In HIV- individuals, the proportion of β7+ CD4+T cells was higher in ileum compared to either blood or rectum (Fig. 5A), while the proportion of CCR4+ CD4+T cells was lowest in the ileum (5E). The proportion of CD4+T cells that express CXCR3 was higher in both gut sites compared to blood and highest in the rectum (5C). Similar trends were observed for CD8+T cells (5B,D,F).

Comparison of CCR6 expression between gut and blood was confounded by subsequent validation experiments suggesting that collagenase treatment may reduce the proportion of T cells that stain for CCR6. Compared to HIV- participants, HIV+ participants had a trend towards more CXCR3+ CD4+T cells in the blood (5C; p = 0.065). No differences between HIV- and HIV+ individuals were observed in β7+ or CCR4+ CD4+T cells in blood, or in the proportions of β7+, CXCR3+, or CCR4+ CD4+T cells in either gut site.

For CD8+T cells, HIV+ individuals had a lower average proportion of CXCR3+ cells in all sites, though statistically significant results were observed only for the rectum (5D). Similarly, HIV+ individuals tended to have fewer CCR6+CD8+T cells in all three sites, with significant results obtained for the blood (p = 0.007) and rectum (p = 0.028). No significant differences were observed in β7+ or CCR4+ CD8+T cells.

### Correlations

In HIV+ but not HIV- participants, the CD4% in the ileum correlated with that in the rectum (r = 0.61, p = 0.012), while no correlation was observed between either gut site and blood in HIV+ or HIV- participants. In HIV+ individuals, no correlation was observed between the CD4% in either gut site and the proportion of CD4+ or CD8+T cells in gut or blood that expressed CD38, HLA-DR, or both. In HIV+ but not HIV- participants, a strong correlation was observed between the CD4% in the ileum and the proportion of ileal CD4+T cells that express CTLA-4 (r = 0.99, p = 0.0004), with a similar trend in the rectum and an inverse trend in the blood. In HIV+ but not HIV- individuals, there was a trend towards a correlation between CD4% in the rectum (but not ileum) and the proportion of blood CD4+T cells that express β7 (r = 0.75, p = 0.066) or CCR6 (r = 0.75, p = 0.066) but not CXCR3.

## Discussion

In order to examine the normal variation in T cell frequencies and phenotypes in blood and different regions of bowel, and to investigate the degree to which ART can restore normal levels in each site, we measured CD4+ and CD8+T cell numbers and phenotypes in the blood, terminal ileum, and rectum of ART-suppressed HIV+ patients and HIV- comparators. Most measurements of T cell numbers were made using flow cytometry, which allows simultaneous assessment of many different markers. In HIV-uninfected individuals, both gut sites had a lower proportion of CD4+T cells (CD4%) and a higher proportion of CD8+T cells (CD8%) than the blood, though the rectum had a larger CD4% than the ileum and the ileum had a larger CD8%. Similar trends were seen for the ratio of T cells to CD45+ leukocytes and to total cells, though comparisons between blood and gut are less meaningful because Ficoll isolation excludes most neutrophils from the PBMC, while these cells are included in the CD45+ population from the gut.

Despite suppression with ART for years, HIV+ individuals had lower average CD4+T cell frequencies in all 3 sites, although there appeared to be differences between measures and sites. In the blood and ileum, HIV+ individuals had a lower average CD4% and CD4+T/total cells, suggesting lower relative and absolute CD4+T cell numbers, whereas in the rectum, they had a lower average CD4% but no difference in CD4+T/total cells, suggesting lower relative but not absolute CD4+T cell numbers. Similarly, the ratio of the mean CD4% in HIV+ to HIV- individuals (HIV+/HIV-) was considerably greater in rectum than either ileum or blood, suggesting less depletion or greater reconstitution in the rectum. This finding is supported by prior studies suggesting greater CCR5+CD4+T cell depletion in the ileum compared to colon[22], less restoration in the duodenum compared to colon[25], and possible restoration of normal CD4% in the rectum[17].

HIV-mediated differences in CD8+T cell numbers also appeared to differ between sites. In the blood, HIV+ individuals had a higher average CD8% and CD8+T/total cells, suggesting higher relative and absolute CD8+T cell numbers, whereas in the ileum, HIV+ individuals had a higher average CD8% but no difference in CD8+T/total cells, suggesting higher relative but not absolute CD8+T cell numbers. In the rectum, no significant difference was observed in either relative or absolute CD8+T cell frequencies.

While flow cytometry allows for simultaneous measurement of multiple markers, it does not provide true data on absolute cell numbers, since the relative proportion of CD4+T cells can be decreased by increases in other cell types. In one study comparing both techniques, HIV+ patients had an abnormally low CD4% in the ileum and colon by flow cytometry but normal absolute CD4+T cell numbers by immunohistochemistry[21]. Therefore, we measured absolute CD4+T cell numbers in a subset of ileal biopsies using immunohistochemistry. Though the sample size was inadequate to assess CD4+T cell numbers in the lymphoid aggregates (LA), our data suggest there may be persistent depletion in the lamina propria (LP; effector site). This finding is consistent with other reports showing that acute HIV infection results in greater CD4+T cell depletion in the LP[6,9,37] and that ART normalizes CD4+T cell numbers in the organized lymphoid tissue but not the LP of the rectosigmoid[11].

As a first step to investigate the phenotype of T cells in different sites, flow cytometry was used to measure the relative frequencies of different T cell maturation subsets. In HIV- individuals, we found striking differences between sites. Whereas naïve cells constituted the largest proportion of both CD4+ and CD8+T cells in the blood, both gut sites had predominantly effector and transitional memory cells, with very low percentages of either naïve or terminally-differentiated cells, suggesting marked differences in the origin or maturation of gut T cells. Our ability to detect differences between HIV- and HIV+ individuals was limited by the relatively low number of individuals analyzed with panels 2 and 3. Nevertheless, we found that HIV+ individuals had a lower average proportion of naïve CD4+T cells in the blood, in agreement with prior reports. Though terminally-differentiated (TD) effector cells constitute a relatively small proportion of all gut T cells, we also discovered that HIV+ patients have a lower proportion of TD CD4+T cells in both gut sites and fewer TD CD8+T cells in the ileum. Assuming that these TD cells are relatively short-lived cells that differentiate from memory cells, the lower frequencies in HIV+ patients could represent a selective block to differentiation.

To investigate T cell “activation,” we measured CD38 and HLA-DR by flow cytometry. In HIV- individuals, the proportion of CD38+ T cells was much higher in the gut compared to blood and was highest in the ileum. Though CD38 is found on naïve as well as activated T cells, naïve T cells constitute a very low percentage of T cells in the gut, so this fact would not explain and would actually result in underestimation of differences between gut and blood. Similarly, both gut sites had higher proportions of HLA-DR+ and CD38+HLA-DR+ CD4+T cells, suggesting higher levels of CD4+T cell activation, perhaps due to continual exposure to microbial antigens from the gut flora. Despite suppression of HIV with ART, HIV+ individuals had a higher average proportion of activated T cells in both blood and gut, though the signature of activation differed between gut and blood. In both gut sites, the difference between HIV+ and HIV- individuals was limited to the proportion of CD38+ CD4+T cells, whereas in the blood, the difference was driven more by HLA-DR and dual CD38+HLA-DR+ cells and was observed for both CD4+ and CD8+T cells. These findings agree with one prior study reporting a normal proportion of HLA-DR+ T cells in the rectosigmoid[17] but contrast with another study showing a higher proportion of HLA-DR+CD45RO+ CD4+T cells in the rectum[11]; neither studied CD38 or the ileum, and differences could be due to patient factors or methods.

In HIV- individuals, very few CD4+T cells in the blood expressed CTLA-4, whereas the average proportion of CTLA-4+ CD4+T cells was considerably higher in both gut sites. Negative signaling through CTLA-4 could help reduce the consequences of excess T cell activation due to microbial products and could help to induce tolerance to the normal gut flora. Compared to HIV- individuals, HIV+ individuals tended to have a higher proportion of CTLA-4+ CD4+T cells in both gut sites, though the difference reached significance only for the rectum; this finding in the rectum agrees with one prior study[28].

To investigate homing potential and Th phenotype, we also measured expression of β7, CXCR3, CCR4, and CCR6. It should be noted that the integrin β7 can complex with molecules other than α4 (such as αE), so antibodies to β7 are not specific for the α4β7 heterodimer. However, previous reports have cited the fact that most β7 is bound to α4, and have used this to justify measuring β7 as a surrogate for α4β7[38,39]. In HIV- individuals, the ileum was notable for a very high percentage of β7+ CD4+T cells, while the rectum had the highest proportion of CXCR3+ CD4+T cells. Since the HIV Env binds to α4β7 and HIV DNA levels have been found to be higher in peripheral CD4+T cells expressing CXCR3, higher gut expression of α4β7 and CXCR3, along with CCR5 and CXCR4, may contribute to the higher observed frequency of infected CD4+T cells in the gut[30].

Though correlations do not indicate causality, we assessed for correlations between CD4+T cell frequencies and factors that might logically be expected to impact immune reconstitution (T cell activation, anergy, and gut homing) in order to generate hypotheses about possible mechanisms. In contrast to others, we found no correlation between the CD4% in the blood and either gut site, which could reflect a lack of power or differences in the mechanisms that govern relative CD4+T cell proportions in the blood and gut. However, we did observe a correlation between the CD4% in the ileum and rectum of HIV+ individuals, suggesting there are some similarities in the factors that affect CD4+T cell depletion or reconstitution in these two sites. While other studies have reported a negative correlation between the proportion of circulating CD38+T cells and absolute CD4 counts in blood[40,41,42], we detected no correlation between CD4% in either gut site and the proportion of CD38+ or HLA-DR+ CD4+ or CD8+ T cells in gut or blood, which may suggest that T cell activation, at least as measured by these markers, is not the major factor impeding immune reconstitution in the gut. In contrast, we found a very strong, direct correlation between the CD4% in the ileum and the proportion of ileal CD4+T cells that express CTLA-4. CTLA-4 is a negative T cell regulator and anergy marker that is overexpressed on regulatory T cells[43,44] and HIV-specific T cells[45]. If CTLA-4 expression impacts relative CD4 numbers, it could be that decreased responsiveness to T cell stimulation, a higher frequency of regulatory T cells, and/or a higher frequency of HIV-specific (though possibly less responsive) CD4+T cells favors relative CD4 reconstitution in the ileum.

In contrast to other studies that have reported a relative depletion of circulating β7+ T cells in HIV+ patients[46], we detected no depletion (relative to HIV- individuals) in the proportion of CD4+T cells in blood or either gut site that express the gut homing markers β7, CXCR3, or CCR6. Subject to the caveats of low sample size and the effects of collagenase on CCR6, this finding may suggest that the residual differences in CD4% are not completely explained by selective downregulation of gut homing markers or depletion/redistribution of gut-homing cells. At the same time, we did observe a trend towards a correlation between the CD4% in the rectum and the proportion of circulating CD4+T cells that express β7 and CCR6, suggesting that the expression of these homing receptors may be one factor that impacts relative CD4 reconstitution in the rectum. This finding agrees with others who have found a correlation between β7+ cells in the blood and relative as well as absolute CD4+T cell counts in the gut[21].

Additional study limitations should be acknowledged. Given the prevailing demographics and methods of sampling, most gut biopsies were obtained from men over age 50, and we were limited in the ability to control for differences between the HIV- and HIV+ populations. Like many recent studies, we focused on ART-treated HIV+ patients and did not have prospective samples or an untreated HIV+ group, which limits the ability to determine the degree to which differences between HIV- and HIV+ groups reflect the impact of the original HIV infection versus partial restoration on ART. While the number of study participants was large relative to most prior studies of human gut tissue, factors such as the occasional inability to intubate the ileum, sequential development of flow panels, and the loss of samples for IHC may have limited the power to find differences between sites or groups and the interpretation of negative (but not positive) results.

Results from this study suggest that different mechanisms may contribute to T cell reconstitution, including homing of cells to the rectum and mucosal anergy in the ileum. Additional research is needed to understand the normal gut immune system and the mechanisms that govern HIV-mediated CD4+T cell depletion and ART-mediated immune reconstitution in different effector and central lymphoid tissues. Many potentially relevant factors were not assessed in this study, including abortive infection leading to pyroptosis[47], ongoing replication despite ART[20], inflammation due to HIV antigens (even in the absence of ongoing replication) or microbial translocation, fibrosis[48,49], and local homeostatic proliferation. An understanding of these mechanisms may help to devise new therapies that can restore gut immune function, which could be critical for reducing gut permeability, systemic immune activation/inflammation, end organ damage, and morbidity/mortality that persist despite treatment of HIV.

## Supporting Information

S1 Fig Gating strategy for analysis of T cell CTLA-4 expression and maturation markers. CD4+ and CD8+ T cells from both PBMC and gut samples were identified as shown in panel A by gating on single cells, CD45+ cells and live (Aqua Amine Reactive Dye negative) CD3+ cells before gating on CD4+ and CD8+ cells. Fluorescence minus one controls were performed on PBMC for each sample to set the CTLA-4 and maturation marker gates (not shown). Gating for CTLA-4 expression on CD4+ T cells is shown in panel B for PBMCs (left) and ileum (right). Maturation markers CD45RO, CD27 and CCR7 were used to identify naïve (Naïve: CD45RO-CD27+CCR7+), terminally-differentiated (Td: CD45RO-CD27-CCR7-), central memory (CM:CD45RO+CD27+CCR7+), transitional memory (TM:CD45RO+CD27+CCR7-), effector memory (EM:CD45RO+CD27-CCR7-), and other memory (OM: CD45RO+CD27-CCR7+) for CD8+ (panels C and D) and CD4+ (panels E and F) T cells. Gating is shown for PBMC (panels C and E) and ileum (panels D and F).
(TIFF)

S2 Fig Gating strategy for identifying T cell activation, β7 integrin, CCR4, and CXCR3. A-B: CD4+ and CD8+ T cells from both PBMC and gut identified by gating on single CD45+ CD3+ cells. Gating of activated (CD38+HLADR+) T cells is shown for PBMC (panel A) and ileum (panel B) on both CD8+ (left) and CD4+ (right) T cells. Fluorescence minus one controls were performed on PBMC for each sample to set the activation marker gates (not shown). C-F: CD4+ and CD8+ T cells from PBMC and gut were identified as described in Fig. 1 and gated for expression of β7 integrin, CCR4 and CXCR3 on CD8+(panels C and D) and CD4+ (panels E and F). Gating is shown for PBMC (panels C and E) and ileum (panels D and F). Fluorescence minus one controls were performed on PBMC for each sample to set the β7 integrin, CCR4 and CXCR3 gates (not shown).
(TIFF)

S3 Fig Absolute CD4+T cell numbers in ileum. A: Photomicrograph after immunohistochemical staining for CD4 (brown) in ileum of representative HIV uninfected individual (left) and HIV+ participant (right); the red boxed insets indicate the area that is magnified relative to low power; scale bar equals 100 microns. B-C: Absolute CD4+T cell numbers, as measured by immunohistochemistry, in lamina propria (B) and lymphoid aggregates (C) of ileum in HIV- (open squares) and HIV+ (black squares) participants. Bars indicate the mean.
(TIFF)
