# Supplementary figures and images for: Site-Specific Differences in T Cell Frequencies and Phenotypes in the Blood and Gut of HIV-Uninfected and ART-Treated HIV+ Adults
Source: PLoS One. 2015 Mar 26;10(3):e0121290. doi: 10.1371/journal.pone.0121290 (PMC4374729; doi:10.1371/journal.pone.0121290)

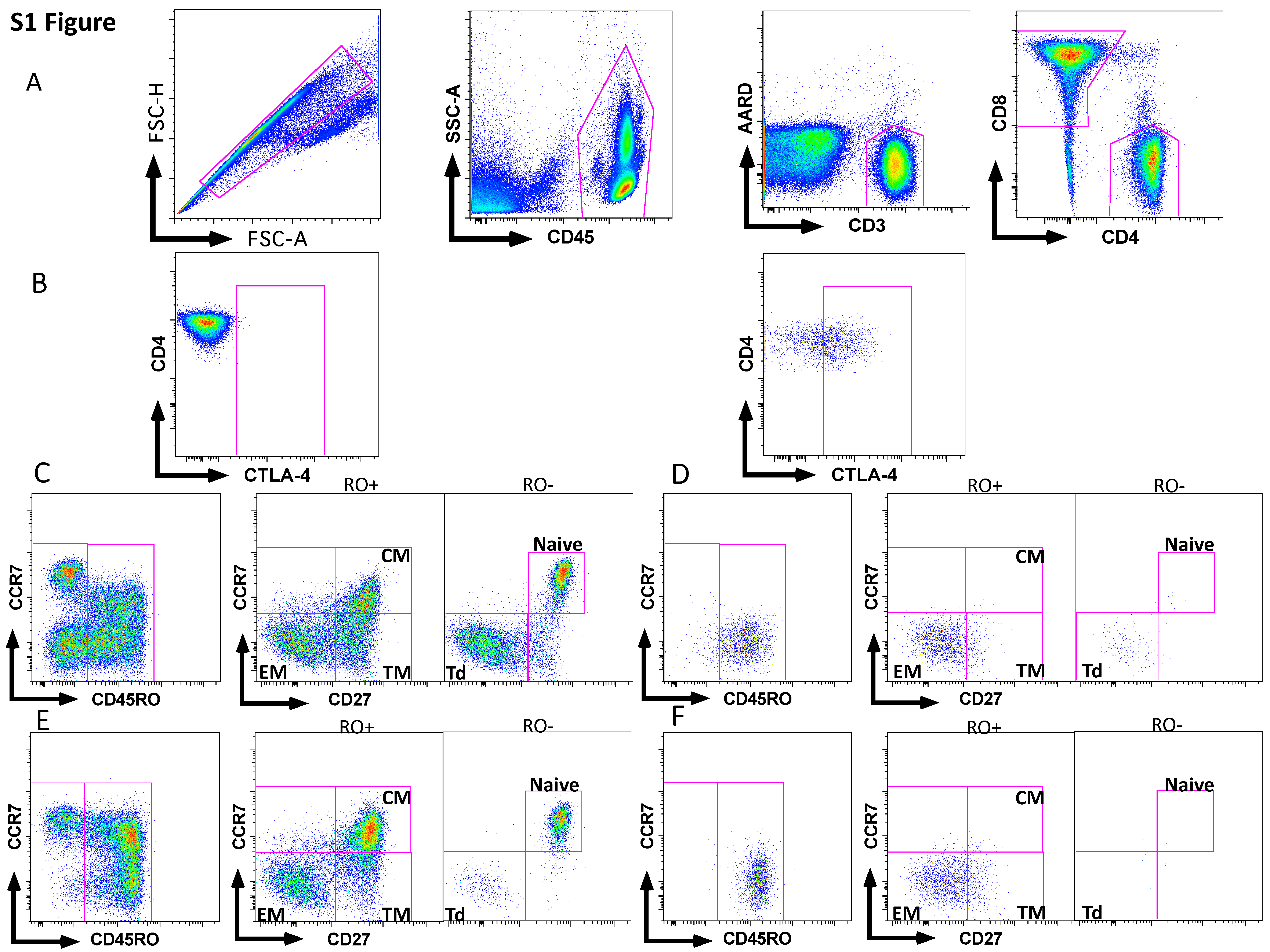

Supplement: S1 Fig — CD4+ and CD8+ T cells from both PBMC and gut samples were identified as shown in panel A by gating on single cells, CD45+ cells and live (Aqua Amine Reactive Dye negative) CD3+ cells before gating on CD4+ and CD8+ cells. Fluorescence minus one controls were performed on PBMC for each sample to set the CTLA-4 and maturation marker gates (not shown). Gating for CTLA-4 expression on CD4+ T cells is shown in panel B for PBMCs (left) and ileum (right). Maturation markers CD45RO, CD27 and CCR7 were used to identify naïve (Naïve: CD45RO-CD27+CCR7+), terminally-differentiated (Td: CD45RO-CD27-CCR7-), central memory (CM:CD45RO+CD27+CCR7+), transitional memory (TM:CD45RO+CD27+CCR7-), effector memory (EM:CD45RO+CD27-CCR7-), and other memory (OM: CD45RO+CD27-CCR7+) for CD8+ (panels C and D) and CD4+ (panels E and F) T cells. Gating is shown for PBMC (panels C and E) and ileum (panels D and F). (TIFF) [file pone.0121290.s001.tiff]

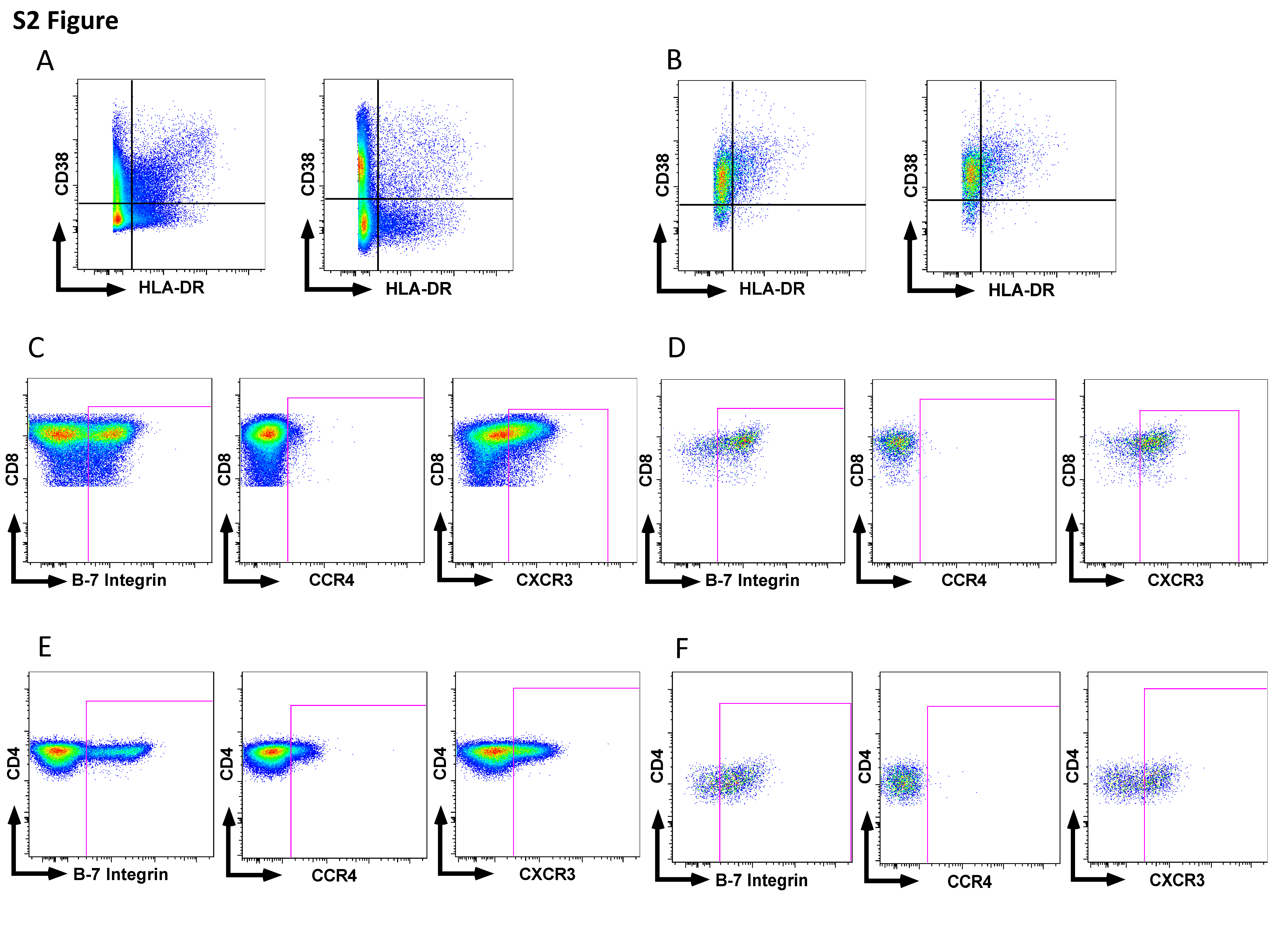

Supplement: S2 Fig — A-B: CD4+ and CD8+ T cells from both PBMC and gut identified by gating on single CD45+ CD3+ cells. Gating of activated (CD38+HLADR+) T cells is shown for PBMC (panel A) and ileum (panel B) on both CD8+ (left) and CD4+ (right) T cells. Fluorescence minus one controls were performed on PBMC for each sample to set the activation marker gates (not shown). C-F: CD4+ and CD8+ T cells from PBMC and gut were identified as described in Fig. 1 and gated for expression of β7 integrin, CCR4 and CXCR3 on CD8+(panels C and D) and CD4+ (panels E and F). Gating is shown for PBMC (panels C and E) and ileum (panels D and F). Fluorescence minus one controls were performed on PBMC for each sample to set the β7 integrin, CCR4 and CXCR3 gates (not shown). (TIFF) [file pone.0121290.s002.tiff]

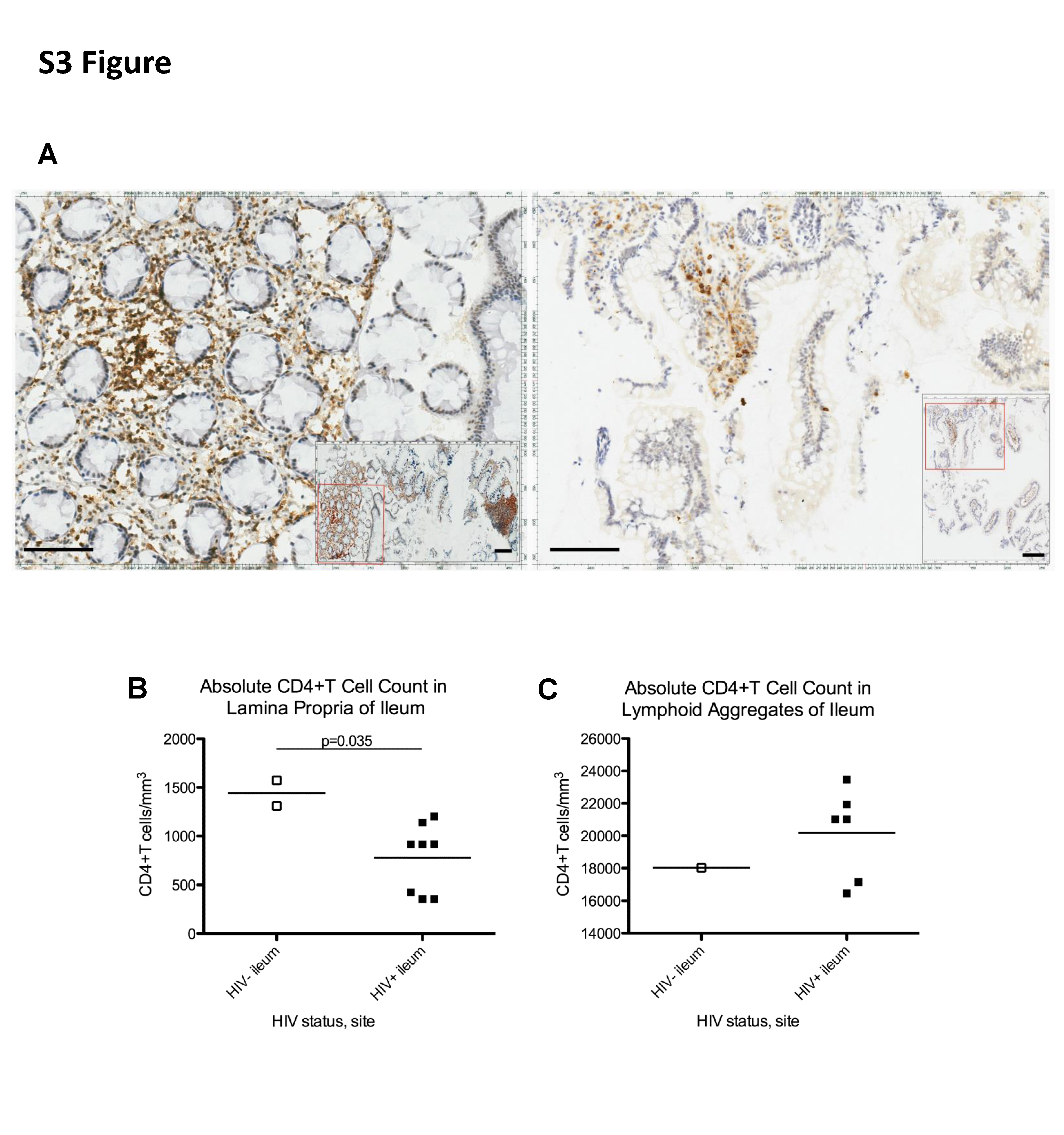

Supplement: S3 Fig — A: Photomicrograph after immunohistochemical staining for CD4 (brown) in ileum of representative HIV uninfected individual (left) and HIV+ participant (right); the red boxed insets indicate the area that is magnified relative to low power; scale bar equals 100 microns. B-C: Absolute CD4+T cell numbers, as measured by immunohistochemistry, in lamina propria (B) and lymphoid aggregates (C) of ileum in HIV- (open squares) and HIV+ (black squares) participants. Bars indicate the mean. (TIFF) [file pone.0121290.s003.tiff]
